# Modeling Epidemics with Dynamic Small‐World Networks

**DOI:** 10.1063/1.1985392

**Published:** 2005-06-21

**Authors:** Kimmo Kaski, Jari Saramäki

**Affiliations:** * Laboratory of Computational Engineering, Helsinki University of Technology, P.O. Box 9203, FIN‐02015 HUT, Finland; University of Aveiro; University of Aveiro; University of Aveiro; University of Aveiro; University of Aveiro

## Abstract

In this presentation a minimal model for describing the spreading of an infectious disease, such as influenza, is discussed. Here it is assumed that spreading takes place on a dynamic small‐world network comprising short‐ and long‐range infection events. Approximate equations for the epidemic threshold as well as the spreading dynamics are derived and they agree well with numerical discrete time‐step simulations. Also the dependence of the epidemic saturation time on the initial conditions is analysed and a comparison with real‐world data is made.

## INTRODUCTION

Modeling the process of disease spreading has traditionally relied on differential equations, describing the dynamics of infection spreading within uniformly mixed populations [1, 2, 3]. The basic premise of uniform mixing is that contacts between all individuals in the population are equally likely, and thus any infected person is equally likely to infect any other person. The spreading process itself is modeled using rate equations, describing population flows between epidemiological classes of individuals, such as susceptible (S), exposed (E), infective (I), and recovered (R). The simplest of these types of models is the widely-utilized Susceptible-Infected-Removed (SIR) model [see e.g. 2, 4], in which susceptible individuals (S) may become infected (I) and continue to infect others until finally removed (R) from the population due to recovery, death, or containment. In this approach, however, the uniform mixing hypothesis has long been considered unrealistic, and spatial effecs and heterogeneity have been shown to profoundly affect disease transmission, persistence and evolution [5, 6, 7, 8, 9, 10]. Instead of relying on this kind of mean-field models and due to recent advances in the research of complex networks [11, 12, 13], there has been increased interest in trying to capture the effects of contact patterns between individuals. These patterns can be described by *contact networks*, where the vertices correspond to individuals and the edges to contacts between them [14]. One of the main motivations for studying complex networks has been to better understand the structure of *social networks* [15, 16], which without doubt has to be reflected in contact networks. Thus there is a natural link between epidemic modeling and research of complex networks.

Utilizing complex networks as spreading lattices in epidemic models has provided much insight in the context of human disease, as well as electronic viruses [15, 9, 17, 18, 19, 20, 21, 22]. A key result of these studies is that spreading processes are very

CP776, *Science of Complex Networks: From Biology to the Internet and WWW; CNET 2004,* edited by J. F. F. Mendes, S. N. Dorogovtsev, A. Povolotsky, F. V. Abreu, and J. G. Oliveira
© 2005 American Institute of Physics 0-7354-0262-0/05/$22.50

fast on realistic network topologies, especially on *small-world* and *scale-free* networks, due to the effect of very short average vertex-to-vertex distances and, in the case of scale-free networks, due to the presence of highly connected hubs. Here, we discuss a "minimal" model of the spreading dynamics of disease transmitted by random contacts, such as common influenza [23]. The basic idea is to capture some salient features of spreading dynamics by utilizing the SIR mechanism on a dynamically changing small-world contact network[1]. Related works on static and dynamic small-world models [15, 24, 25, 26, 27, 28, 29] have shown the existence of a shortcut-density-dependent epidemic threshold as well as scaling properties. In contrast to these studies, our focus is explicitly on the spreading dynamics, i.e., on the development of an epidemic, as formulated in terms of rate equations.

This presentation is organized such that first we describe the spreading model in terms of a probabilistic discrete time-step process. Next, we address the issue of the epidemic threshold and derive necessary conditions for the epidemic to start spreading. This is followed by the derivation of analytical equations for a special case of this model, where the probability of infection transmission between neighboring infective and susceptible individuals is set to one. Then we discuss the dependence of the duration of an epidemic on the initial conditions and the system size, and compare the time series generated with the model to real-world data. Finally, we summarize the results of this study.

## DISCRETE TIME-STEP SIR MODEL ON DYNAMIC SMALL-WORLD NETWORK

A key ingredient of our model is the contact network, through which the infection spreading takes place. Like social networks various contact networks display the *small-world* property, which means that long-range contacts between individuals result in short average distances along the edges of the network. In epidemiology these long-range contacts can be considered either infrequent contacts or random encounters taking place in an underlying regular short-range network structure, which in turn can be interpreted as groups of people having regular or frequent contacts – such as those between friends or colleagues. In order to capture these features, we define the contact network as comprising a regular one-dimensional lattice of $N$ vertices with fixed coordination number $2z$, and with additional temporary long-range links, changing their configuration at random. It is noted that periodic boundary conditions are assumed, as depicted in Fig. 1. This network acts as the substrate for disease spreading, composed of two different processes: the short-range (SR) process corresponding to passing the disease on to the nearest circle of persons, i.e. along the underlying regular lattice, and the long-range (LR) process to infecting any randomly encountered person.

The main dynamics of spreading is modeled using the SIR mechanism, such that individuals in the network are at any given time labeled as being either susceptible (S),

[1] In terms of population mixing, one can view small-world networks analoguous to a situation where there are clusters of densely connected individuals (e.g. social groups), having contacts with "far-off" groups through sparse long-range links.

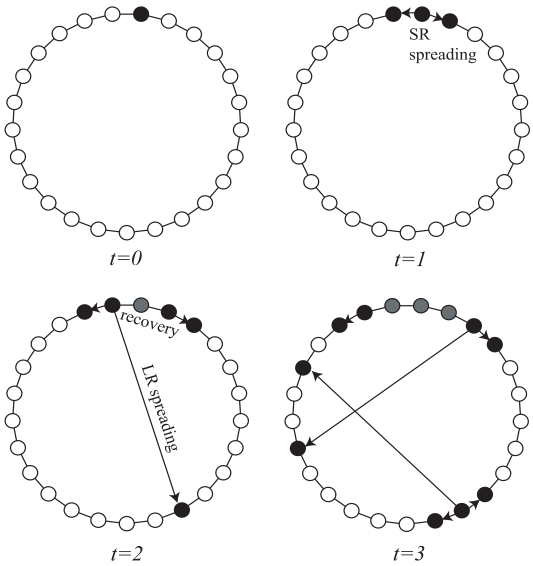

**FIGURE 1.** Schematic of epidemic spreading on a dynamic small-world contact network with coordination number $2z = 2$. At time $t = 0$, a single vertex is infected (solid black circle). Then the infection spreads to neighboring vertices ($t = 1$) as well as randomly chosen far-off vertices ($t = 2, t = 3$). Recovered vertices are shown as solid grey circles.

or infected (I) or recovered (R). Initially, the number of individuals being suceptible is set to $N - N_0$ with $N_0$ being the number of infected individuals. The dynamics of the model is such that at every discrete time step of duration $\Delta t$, each infected individual in the network

1. Infects its nearest neighbors, if susceptible, so that each infection occurs with probability $p_s$,
2. With probability $p_j$ an infected individual tends to infect one randomly chosen far-off individual, succeeding if the individual is susceptible,
3. With probability $p_r$ the individual recovers and can no longer be infected or infect others.

This process can be readily iterated numerically until changes no longer take place. The process step (1) corresponds to transmission of infection along the regular underlying lattice (the short-range process) and step (2) amounts to the randomly changing long-range connections (the long-range process).

## ANALYTICAL DESCRIPTION

### Epidemic threshold

Let us first derive the necessary conditions for an epidemic to take off, i.e. the epidemic threshold, based on analysis of rate equations describing the dynamics of the system. For simplicity, we will set $2z = 2$, so that each network vertex or individual has two nearest neighbors. Due to the fact that the spreading process is stochastic, we will describe its characteristics with average quantities, which should be interpreted as averages over several epidemics. By $I(t)$ we denote the average number of infected individuals at time $t$ and $N'(t)$ the average number of individuals being ever infected. We also define an auxiliary quantity $F(t)$, which denotes the average number of pairs of vertices where one vertex is infected and its nearest neighbor susceptible, i.e., the number of fixed edges along which a short-range transmission can happen. Here we call $F(t)$ as the amount of domain walls, such that the short-range spreading process can be viewed as growth of domains of infected people with the walls of these domains moving with average velocity $p_s$. Using this auxiliary quantity the rate equations for $N'(t)$ and $I(t)$ can be written as:

$$\frac{dN'(t)}{dt} = p_s F(t), \tag{1}$$

and

$$\frac{dI(t)}{dt} = p_s F(t) - p_r I(t). \tag{2}$$

The domain wall dynamics is as follows: the walls are created by the long-range spreading events, two walls per event. The walls are annihilated by events where an infected individual recovers before transmitting the disease to one of its short-range neighbors, as well as by merging events, where two spreading domains collide and merge. The LR spreading "jumps" originating from the infected individuals occur with probability $p_j$, and succeed if the randomly chosen target vertex is susceptible, the probability of which equals $[N - N'(t)]/N$. Now let us consider a situation where at $t = 0$ we have a small initial number $I_0$ of infected individuals. For early times $t$ and large $N$, we can neglect the effect of domain wall merging. Then, the rate equation for the amount of domain walls can be written as

$$\frac{dF(t)}{dt} = 2p_j \frac{N - N'(t)}{N} I(t) - p_r [1 - p_s] F(t). \tag{3}$$

Here the first term on the r.h.s. corresponds to the creation of spreading domains and the second term to the effect of individuals recovering before disease transmission. At early times $t$, we may approximate that $[N'(t)/N] I(t) \approx 0$. Then, by combining Eqs. (2) and (3) and moving to scaled variables, $i(t) = I(t)/N$, we get

$$\frac{d^2 i(t)}{dt^2} + p_r (2 - p_s) \frac{di(t)}{dt} - \left[2 p_s p_j - (1 - p_s) p_r^2\right] i(t) = 0. \tag{4}$$

This equation has an exponential solution with two real eigenvalues $\lambda_{1,2}$, where the second eigenvalue is always negative. If the first is positive, the epidemic will take off leading to exponential growth of the number of infected. This condition is satisfied if

$$p_j > \frac{p_r^2(1-p_s)}{2p_s}, \tag{5}$$

which illustrates that in our simplified picture a certain probability of long-range jumps is necessary for passing the epidemic threshold. This is closely related the to the corresponding percolation threshold on fixed small-world networks [24].

Figure 2 illustrates the threshold of long-range infection probability $p_{j,c} = p_r^2(1-p_s)/2p_s$ as a function of short-range transmission rate $p_s$ when the average recovery time was fixed to $1/p_r = 6$, (i.e. $p_r = 0.167$). The solid line indicates values given by Eq. (5), and the open squares show results of numerical simulations, which were generated using the discrete-time-step model on networks with $N = 150,000$ individuals and $N_0 = 15$ of them initially randomly infected, and after averaging the maximum epidemic sizes $n_{max} = N'_{max}/N$ over 50 runs. By taking the finite system size into account and following [24], we chose the numerical threshold values $p_{j,c}$ as the points where $n_{max}$ exceeds 0.2.

Next we make a note concerning the basic reproductive number $R_0$, which is defined as the average number of secondary infections produced by one infected individual introduced into a susceptible population. This quantity describes the epidemic threshold such that an epidemic will take off only if $R_0 \geq 1$. It should be noted that the spreading dynamics is often seen to depend mainly on $R_0$. However, it is well known that this quantity is not useful outside the uniform mixing assumption, and corrections have been suggested to take it into account in more complex spreading models [see e.g. 2]. In our case, the spreading dynamics cannot be solely determined by the number of secondary infections caused by an infected individual. The reason is that the effect of a secondary infection caused by nearest-neighbor transmission is different from the one caused by a long-range jump. Especially in the initial stages of the disease, long-range jumps will (almost) always generate new spreading domains expanding with the rate $2p_s$ (or $\sim 2p_s z$ for larger neighborhoods), whereas nearest-neighbor transmissions only expand existing domains.

## Dynamics in the limit of $p_s = 1$

Next we focus on considering the situation where nearest-neighbor spreading probability $p_s$ approaches unity, which means that the nearest neighbors will always become infected before recovery takes place. In this case, the epidemic will eventually cover the whole network of individuals, which makes the analytical treatment of the dynamics of the model tractable. In the model the assumption that the network represents the whole susceptible population, is not quite realistic. However, we can conjecture that a model where we *a priori* define the vertices of the network to represent only those individuals who at some point *will become* infected represents the spreading dynamics adequately. In this case, the quatity $N$ represents the total epidemic size rather than the entire suscep-

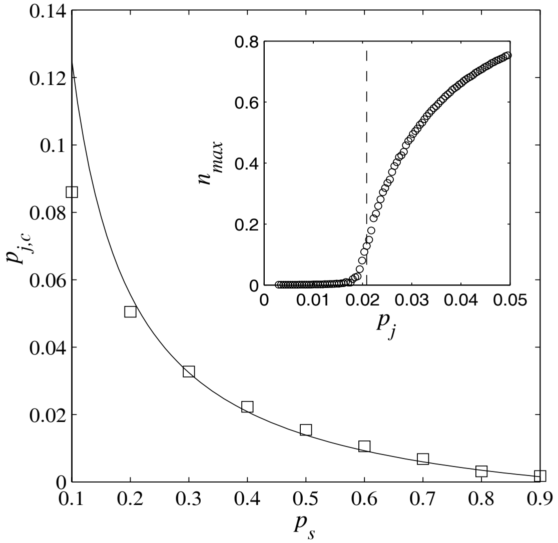

**FIGURE 2.** Numerical results for the threshold of long-range probabilities $p_{j,c}$ (□) as a function of short-range transmission rate $p_s$ for the average recovery time $1/p_r = 6$ (i.e. $p_r = 0.167$) in networks of size $N = 150,000$, and with $N_0 = 15$. The solid line indicates an analytic approximation to $p_{j,c}$. Inset: an example simulated data series, displaying the average maximum epidemic size $n_{max}$ (○) as function of $p_j$ for $p_s = 0.4$. The dashed line indicates the analytic approximation for the threshold.

tible population size. The rate equations for the spreading dynamics with $p_s = 1$ can be now derived utilizing the domain wall approach discussed above; Eqs. (1) and (2) still hold in the present framework, and we only have to rewrite the equation describing the domain wall dynamics. Despite our assumption that $p_s = 1$, we will keep the parameter in the formulas for reasons discussed below.

In the $p_s = 1$ limit, the rate equation for the amount of domain walls needs to be modified as follows: the domain wall creation term in Eq. (3) remains the same, but now the walls are destroyed by domain merging alone. Hence, we need a term describing the rate of this annihilation process. At any given time, the network contains $F(t)/2$ connected domains of susceptibles, containing altogether $N - N'(t)$ susceptible individuals. These domains "shrink" with velocity $2p_s$ due to the moving walls and the average domain length is $2[N - N'(t)]/F(t)$, with the average lifetime of $[N - N'(t)]/[F(t)p_s]$. Two walls are destroyed through collision, and since there are $F(t)/2$ domains, then on the average $p_s F(t)^2/[N - N'(t)]$ walls will be destroyed in unit time. Combining the above, we arrive at

$$\frac{dF(t)}{dt} = 2p_j \frac{N - N'(t)}{N} I(t) - p_s \frac{F(t)^2}{N - N'(t)}. \tag{6}$$

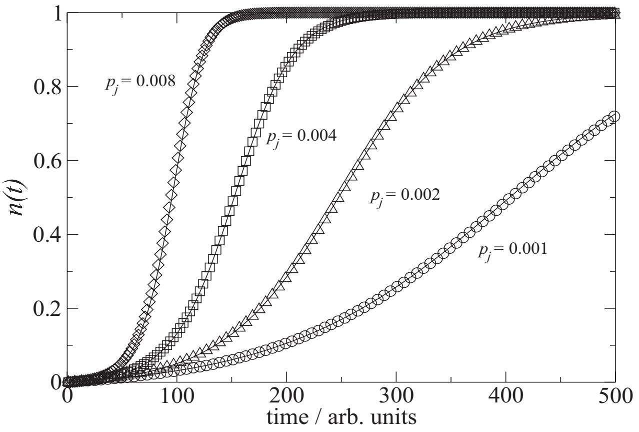

**FIGURE 3.** The fraction of ever infected vertices $n$ as function of time $t$ calculated by simulated spreading on $N = 250,000$ networks for different long-range jump probability $p_j$ values (diamonds, squares, triangles, circles) and by numerical integration of Eqs. (7) and (8) (solid lines), with nearest-neighbor spreading rate $p_s$=1, recovery rate $p_r$=0.17. The results are averages over 100 runs.

Moving to scaled variables $i(t) = I(t)/N$ and $n(t) = N'(t)/N$ and combining Eqs. (1), (2) and (6) yields

$$\frac{d^2n(t)}{dt^2} + \frac{[dn(t)/dt]^2}{1-n(t)} = 2p_sp_j\,[1-n(t)]\,i(t), \tag{7}$$

$$\frac{di(t)}{dt} = \frac{dn(t)}{dt} - p_ri(t). \tag{8}$$

The system defined by Eqs. (7) and (8) can be solved by numerical iteration for both $n(t)$ and $i(t)$ using standard methods.

In Figure 3 we show the results of numerical iteration and also the results of discrete time-step simulations, averaged over 100 runs. In these simulations, the network size was set to $N = 250,000$, the recovery probability per unit time to $p_r = 0.17$, and the spreading velocity to $p_s = 1$, while the long-range jump probability was varied. The initial fraction of infected individuals was set to $n_0 = 25/250,000 = 10^{-4}$. The fraction of individuals ever infected $n(t)$ is seen to follow an s-shaped curve such that in the beginning the fraction of infected increases exponentially but then slows down to almost linear increase, as there is less and less susceptible population to be infected. Finally the curve saturates as the epidemic covers the entire susceptible population. From this figure it is seen that the theoretical curves calculated by numerically iterating the above equations match the simulated results very well.

Finally it should be noted that when $p_s < 1$, Eqs. (7) and (8) still approximate the system dynamics quite well, as long as $p_s$ is large enough to justify neglecting the effect of recovery-induced stopping of domain walls. Furthermore, should we wish to consider larger neighborhoods, we can simply absorb the coordination number into the definition of $p_s$ such that $p_s \to p_s z$.

## SCALING OF THE EPIDEMIC SATURATION TIME

It is noteworthy that Eqs. (7) and (8) indicate that the system size $N$ enters the dynamics of the model only indirectly, in the form of the initial conditions $n_0 = N'(0)/N$. Therefore, it is interesting to investigate the duration of the epidemic for different size networks as a function of the initial density of infected $n_0$. Recently, it was shown that in a model where spreading takes place on small-world networks with dynamic rewiring of links and initial density of infected fixed to $n_0 = 1/N$, the epidemic saturation time depends on the network size as $\log N$ [29].

In the types of models presented here, setting the initial conditions is a bit problematic, because in this framework it is impossible to trace the start of an epidemic to a single infected individual. Therefore, we define the starting time of the epidemic in terms of an initial density of infected, such that at $t = 0$, $n(0) = n_0$, $n_0 \ll 1$. Furthermore, we define the saturation time $t_f$ so that $n(t = t_f) = n_f$, where we set $n_f$ to a value less than unity. This "renormalization procedure" takes care of the asymptotic nature of the process both at its beginning and end.

In Fig. 4(a) we show a log-linear plot of the saturation time $t_f$ as function of $n_0$ for several network sizes $N$, ranging from 100,000 up to 750,000. The $N_0$ initially infected vertices were chosen randomly. Here, $t_f$ was set as the time when 95 % of the susceptible population has become infected, i.e. the first time step when $n(t) \geq 0.95$. The results of the simulations are taken as averages over 100 runs each, with $p_j = 0.008$, $p_r = 0.17$ and $p_s = 1$. The solid line depicts a fitted log-linear function of the form, $t_f = -a \times \log n_0 - b$, with $a = 35.8$ and $b = 19.0$, which demonstrates a very good match with the numerical results and thus evidence for $t_f \propto -\log n_0$. This is in line with the $\log N$-scaling observed earlier with $n_0 = 1/N$ [29], since $-\log n_0 = -\log N_0/N$ becomes $\log N$ when $N_0 = 1$.

The independence of the saturation time on the system size is further illustrated in Fig. 4(b), which shows the fraction of individuals ever infected $n(t)$ averaged over 100 simulation runs of epidemics in networks of different size, with the initial condition $n_0 = 1 \times 10^{-4}$ and other parameters being $p_j = 0.005$, $p_r = 0.17$ and $p_s = 1$.

## COMPARISON WITH REAL DATA

In order to verify the validity of our modeling approach, we compare the fraction of currently infected individuals $i(t)$ obtained by numerical iteration of Eqs. (7) and (8) with publicly available data on Influenza A in Finland [30]. The data depicts monthly reported influenza A findings by hospital laboratories during 1997-2002, and can be assumed to linearly correspond to epidemic levels during respective months. Fig. 5 shows the epidemics data during these influenza seasons together with our fitted theoretical curves for

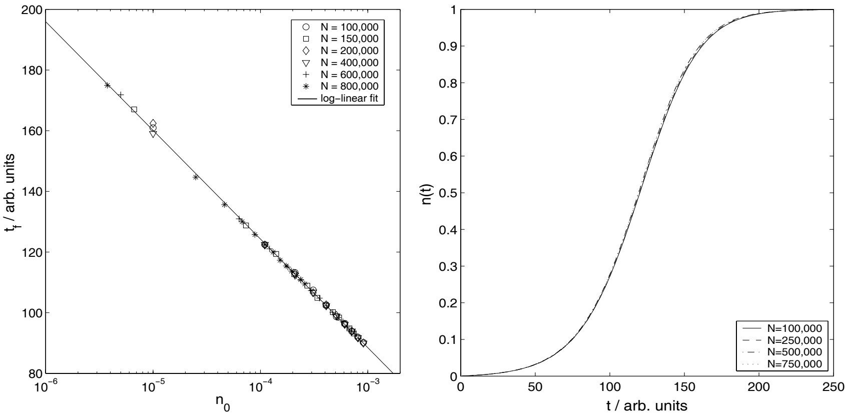

**FIGURE 4.** Left: Saturation time $t_f$ as a function of the initial density of infected $n_0$ for varying network sizes. The solid line displays a log-linear fit, indicating clearly that $t_f \propto -\log n_0$. Right: The average epidemic size $n(t)$ vs. time for several epidemics in networks of different sizes, each started with $n_0 = 1 \times 10^{-4}$, illustrating the independence of the spreading dynamics on the network size when initial conditions are fixed. For other parameters, see text.

$i(t)$ calculated using Eqs. (7) and (8). For these fits, we set $p_r$ = 0.17 / day, which corresponds to an infective period of about 6 days, and the SR spreading probability $p_s$ was set to 1, while $p_j$ was used as the fitting parameter. We first fitted $p_j$ for each individual epidemic and then averaged the results, obtaining $p_j$ = 0.0075 / day as the so called consensus value. Finally, the theoretical epidemic curves were scaled to account for the differing number of susceptibles $N$ for each annual epidemic. The quality of fits using constant spreading parameters indicate that the underlying dynamics can be thought to remain more or less the same for each annual epidemic. Note that fits of similar quality could in principle be obtained by using the equations for the classical fully mixed SIR model [see e.g. 3]; our example merely shows that Eqs. (7) and (8), which were derived starting from more detailed considerations, result in time series which are in accordance with real-world observations.

## SUMMARY

To summarize, we have presented a model for the spreading of randomly contagious diseases such as influenza. The model is based on a stochastic SIR mechanism on dynamic small-world networks, where randomly occurring long-range links are introduced in order to take into account the inherent randomness of spreading. We have derived equations for the epidemic threshold and spreading dynamics, and shown that these match with results of discrete time-step simulations. In addition, we have shown how the epidemic saturation time scales with the system size and initial conditions. Finally, we have compared numerical time series calculated with our model to real-world data and found a good agreement. This presentation is based on our recent publication in Journal of

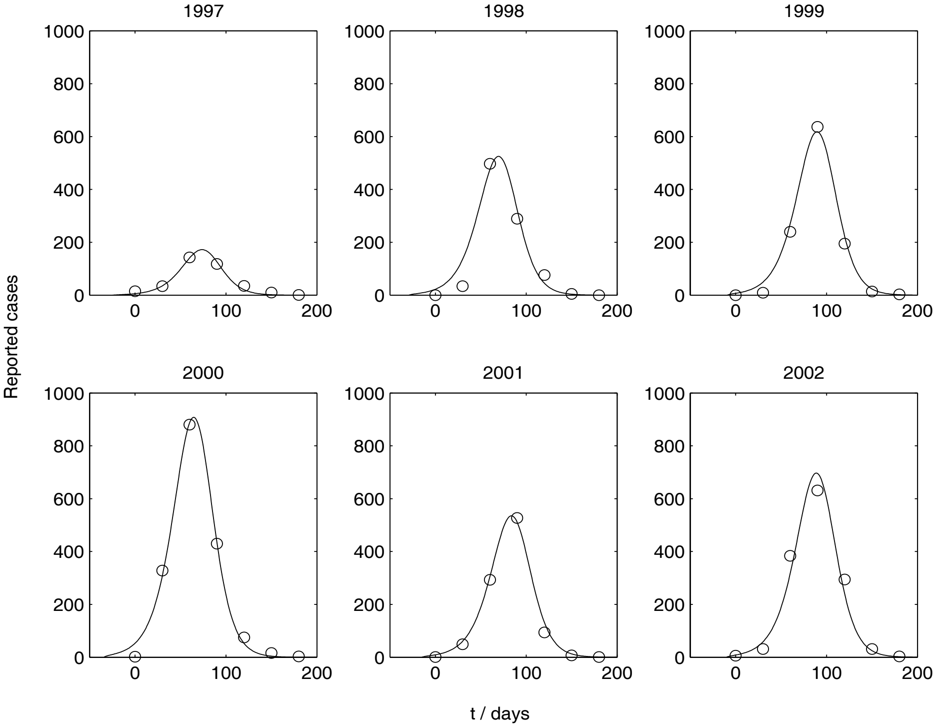

**FIGURE 5.** Monthly laboratory-confirmed cases of Influenza A in Finland (circles) during seasons 1996-1997 to 2001-2002, together with theoretical curves of numbers of infected $i(t)$ calculated using Eqs. (7) and (8), shifting $t = 0$ to correspond for individual epidemics. Spreading parameters for theoretical curves are recovery rate $p_r$=0.17 / day, spreading rate $p_s$=1 / day, long-range jump rate $p_j$=0.0075 / day. The number of infected $i(t)$ has been scaled individually by different $N$ for each annual epidemic.

Theoretical Biology [23], where a more detailed account of this model study and also comparison with some other experimental data sets is presented.

## ACKNOWLEDGMENTS

We thank J.J. Hyvönen and J.-P. Onnela for useful discussions. This work is supported by the Academy of Finland, project No. 1169043 (Finnish Centre of Excellence programme 2000-2005).
